# The Relationship Between Methadone and Buprenorphine Enrollment and Overdose Prevention and Treatment Behaviors Among a Community Sample of People Who Use Opioids in Baltimore, Maryland

**DOI:** 10.3390/ijerph22020213

**Published:** 2025-02-03

**Authors:** Carl A. Latkin, Lauren Dayton, Melissa Davey-Rothwell, Abenaa Jones

**Affiliations:** 1 Department of Health, Behavior & Society, Johns Hopkins Bloomberg School of Public Health, John Hopkins University, Baltimore, MD 21205, USA; ldayton2@jhu.edu (L.D.); mdavey1@jhu.edu (M.D.-R.); 2 Department of Human Development and Family Studies, College of Health and Human Development, The Pennsylvania State University, University Park, PA 16802, USA; avj5462@psu.edu

**Keywords:** overdose, methadone, buprenorphine, harm reduction, prevention, fentanyl

## Abstract

Background: Methadone and buprenorphine can reduce overdose-related mortality. Behavioral approaches can also reduce fatal overdoses. The current study examined the relationship between methadone and buprenorphine and overdose history and overdose prevention and treatment behaviors. Methods: Between December 2022 and August 2024, 647 individuals who used opioids in the prior month enrolled in a community recruited study on overdose. Participants were administered a face-to-face survey. Key behaviors assessed included overdose recency, testing drugs for potency, ingesting drugs slowly, using fentanyl test strips, using drugs alone, and carrying naloxone. Chi-square and logistic regression models examined the relationships between methadone and buprenorphine and overdose-related outcomes. Results: In total, 32.9% of participants were currently taking methadone and 15.5% buprenorphine. Most (69.2%) reported ever overdosing, and among those, 33.7% had overdosed within the prior 6 months. There were no significant associations between methadone or buprenorphine status and overdose prevention and care behaviors. In the multivariable logistic regression model, methadone use was associated with a lower odds ratio (aOR = 0.49, 95% CI = 0.30–0.79of a recent overdose compared to buprenorphine. Daily or almost daily crack use was associated with greater odds of a recent overdose (aOR = 2.21, 95% CI = 1.44–3.39. Discussion: Findings suggest the importance of promoting overdose prevention and care behaviors to people in drug treatment and training them to promote overdose prevention and care behaviors among their drug-using network members and other community members.

## 1. Introduction

Fatal opioid-related drug overdoses continue to be a pressing public health issue, especially in North America, with over 81,000 estimated opioid-related fatalities in 2023 [1]. Medication for opioid use disorder (MOUD) is well documented to reduce mortality associated with overdose as well as all-cause mortality [2,3,4,5,6,7]. It is also established that a substantial proportion of individuals continue to use illicit substances, including opioids, while on MOUD [8,9,10,11,12]. Moreover, most people who use opioids are not on MOUD. A US study from 2015 to 2017 of over 40,000 individuals with opioid use disorder (OUD) found that only 12.5% received methadone or buprenorphine [6]. Additionally, a 2022 study of individuals who perceived that they needed OUD treatment reported that only 25% received buprenorphine, methadone, or extended-release naltrexone [13]. Given this low proportion of people who use opioids on MOUD and the high percentage of people in MOUD who use continue to use drugs, it is critical to promote effective overdose prevention behaviors to people who use drugs in order to reduce opioid-related fatalities. These prevention behaviors include ready access to naloxone; not using drugs alone so that if there is an overdose, naloxone can be administered; and use of methods to assess potency, including fentanyl test strips; testing small amounts of drugs, and slowly ingesting drugs [14,15,16,17].

Albeit effective, MOUD is also not a panacea. Strain et al. reported that at a 30-week assessment, over half the patients on methadone had opioid-positive test results [18]. A community-recruited sample of people who use drugs in Massachusetts found that 39% were currently on MOUD [19]. The authors also reported, using a mixed-methods study design, that insufficient medication dosage, history of trauma, cravings, and contextual triggers lead to drug use while on MOUD. An EMA study of methadone and buprenorphine patients also found that stress-induced cravings predicted substance use [20]. Other research has documented that the intensity of withdrawal symptoms predicts MOUD attrition [21]. This body of research highlights the difficulty in the cessation of opioid use even with the assistance of MOUD. Moreover, although both methadone and buprenorphine substantially reduce mortality, especially overdose mortality, there are periods and situations that heighten risk, including the induction phase for methadone and the time immediately after leaving MOUD; additionally, overdose risk is heightened by illicit opioids being adulterated with highly potent synthetic opioids [21,22].

This opioid overdose risk indicates the importance of training people on MOUD with a set of strategies for overdose prevention and care that will be effective regardless of whether they continue to use illicit opioids or abstain from drug use. Promoting safer drug use strategies may be perceived to conflict with drug treatment goals, which are often cessation of drug use, among some drug treatment providers [23,24]. Although cessation is a goal for many individuals, opioid dependence can be viewed as a chronic and relapsing condition. There are high rates of attrition from drug treatment, which may be due, in part, to non-prescription drug use. In addition to physiological and psychological factors leading to relapse, there is a range of impediments that exist to continuing treatment, including costs, transportation, childcare, and incarceration [12,25].

Two sets of strategies may reduce the risk of a fatal overdose among people who are actively using opioids: engagement in overdose prevention strategies and overdose treatment. In addition to reducing drug use, overdose prevention can involve testing drugs for potency and using small doses. These approaches are critical with fentanyl and other synthetic opioids, which are exceedingly potent. Yet, a sense of time urgency may be a barrier to these strategies due to fear of arrest, lack of privacy, and heightened withdrawal symptoms [26,27,28]. The second strategy of naloxone administration requires immediate access to naloxone and someone to administer it. Ensuring others are available to administer naloxone may be impeded by drug use stigma and distrust due to negative life experiences [23].

The relationship between MOUD and overdose risk and prevention behaviors is not well understood. It may be that those taking methadone and buprenorphine have fewer withdrawal symptoms, and when they do use illicit opioids, they are able to use them in a safer manner. On the other hand, it is possible that those taking methadone and buprenorphine try to hide their illicit opioid use and, hence, use quickly and are then exposed to great overdose risk. The current study examined the relationship between MOUD use, recent overdose, and overdose risk and prevention behaviors in a population of people who currently use opioids.

## 2. Methods

Participants were from the OASIS study, a study that focused on geospatial and social factors and drug overdoses among people who use opioids. They were recruited through community outreach and word of mouth in areas throughout Baltimore City, MD known for high drug use. Enrollment criteria included self-reported illicit opioid use in the prior month, age of 18 years or older, and living in the Baltimore Metropolitan area. Participants were paid USD 50 for the baseline visits. All study protocols were approved by the School of Public Health IRB. Between 7 December 2022 and 13 August 2024, 676 individuals enrolled in the study. The current analyses were restricted to 647 individuals who reported heroin or fentanyl use in the prior month.

### 2.1. Measures 

Measures were adapted from prior studies on overdose prevention behaviors. Five questions assessed overdose prevention behaviors: “How often do you use a small dose first to see how strong your drugs are?”, “How often do you use fentanyl test strips?”, “How often do you go slow to check your drugs strength?”, and “Typically, when you are using, how often do you have Narcan with you?” [16,29]. The response options were “Always”, “Often”, “Sometimes”, “Rarely”, and “Never”. Based on the distributions, these variables were categorized into three groups: always/often, sometimes, and rarely/never. The fifth question, “Typically, when you are using, how often do you have Narcan with you?” was categorized into three groups of “always”, “often/sometimes”, and “rarely/never”.

Frequency of overdose was measured with the questions, “How many times in your life have you overdosed?” and “When was your most recent overdose”, with the response options of “within the past 6 months”, “6 months to one year ago”, and “greater than one year ago”. The categories of 6 months to one year ago and greater than one year ago were combined as the focus of the analyses was on recent overdoses.

To assess drug use, participants were first asked about the last time they used specific drugs (heroin, cocaine, crack, cannabis, buprenorphine, and fentanyl). Those who reported lifetime use were asked about frequency of use in the last three months. The question on buprenorphine stated”, In the past 3 months, how often did you use Buprenorphine/Bupes to get high?” The one on fentanyl was “In the past 3 months, how often did you use fentanyl? This includes using fentanyl alone or other drugs mixed with fentanyl”. Injection drug use in the prior three months was also assessed. Response options included “Never”, “Less than monthly”, “Monthly”, “Weekly”, and “Daily or Almost Daily”. A measure of heroin and fentanyl use was derived by combining the survey items on heroin use and fentanyl use in the past three months into the construct of daily or almost daily fentanyl or heroin use versus less than daily or almost daily use.

Three questions assessed MOUD drug treatment: “Have you ever been in drug treatment”, “When were you most recently in drug treatment”, and “Are you currently taking any of the following medications to treat drug addiction? 1. buprenorphine/suboxone, 2. methadone, 3. Naltrexone, 4. some other medication.

Race was based on self-report to the question, “What race do you consider yourself”. As the study population was almost exclusively (95%) Black and White, all other responses collapsed into the “other” category. Other background variables included employment status, homelessness or unhoused, level of education, diagnosed mental health condition, and incarceration in past 6 months. Sex was defined as sex at birth.

### 2.2. Analyses 

As only 2 individuals reported naltrexone and 8 reported “some other medication”, the analyses focused on buprenorphine and methadone, separately comparing those on buprenorphine and methadone to all others in the sample. Post hoc analyses were conducted with these 10 individuals removed, which did not change the magnitude of the associations. Chi-square and logistic regression models were employed to examine the associations between current MOUD and overdose prevention behaviors. Among the sample of those who had ever overdosed, bivariate and multivariable logistic models assessed factors associated with a recent drug overdose (within the prior six months) compared to those who had overdosed more than six months ago. Multivariable logistic models included demographic variables, homelessness, and frequency of drug use.

## 3. Results

The sample was predominately Black (72.6%) and male (58.9%; Table 1). The mean age of the sample was 50.1, median 53.0, SD = 10.8. The 25th age percentile was 41, and the 75th was 58. A minority reported full or part-time employment (9.5%). Over one-third (43.3%) had been homeless in the prior six months, and over two-thirds (69.7%) reported being diagnosed with a mental health condition such as “depression, bipolar, or schizophrenia”. All participants reported using either heroin or fentanyl in the prior 3 months. Most participants (89.0%) reported heroin use, with 71.1% daily or almost daily use, and 88.4% reported fentanyl use (67.7% daily or almost daily). In addition to fentanyl and heroin, a large (69%) percentage of participants reported crack use, with 44.2% reporting daily or almost daily use. The majority (58.5%) reported cannabis use (23.2% daily or almost daily). Fewer (29.6%) reported injection drug use (19.9% daily or almost daily), 20.4% prescription opioids to get high (7.4% daily or almost daily), and 28.6% power cocaine (8.7% daily or almost daily). Even fewer reported illicit (10.0%) buprenorphine use (1.7% daily or almost daily).

Most (69.2%) participants reported that they had ever overdosed. Among those who reported an overdose, 33.7% had overdosed within the prior 6 months. Regarding overdose prevention and treatment behaviors, less than half (43.0%) reported always having naloxone available when using drugs, and almost half (47.1%) reported always or often using drugs alone. Slightly more than half (55.2%) of the participants reported always or often going slow to check the potency of their drugs, and almost two-thirds (64.3%) reported using a small dose first to assess potency. Most participants (75.1%) reported rarely or never using fentanyl test strips. Less than half (43.0%) reported always having naloxone with them when they used drugs, and 22.4% reported rarely or never having naloxone with them when using.

Slightly more than half (51.6%) of the sample were not taking buprenorphine or methadone, 32.9% reported methadone, and 15.5% buprenorphine. Less than 1% (2 people) reported currently taking naltrexone, and 6.3% (n = 41) reported in-patient or residential treatment. In an analysis of demographic factors and MOUD status, age, gender, education, and homelessness were not associated with MOUD status. However, there was a significant association with race, with 18.1% of Black participants reporting currently taking buprenorphine, compared to 7.7% of White participants, whereas 46.5% of White participants reported currently taking methadone compared to 28.7% of Black participants (Chi-Square = 19.6, *p* < 0.001).

As shown in Table 2a, there were no significant statistical associations between MOUD status and the overdose prevention behaviors of always having naloxone available when using drugs, going slow to check the potency of their drugs, using a small dose first to assess potency, or using fentanyl test strips. The Chi-Square analyses revealed differences in recent overdose rates and drug use frequency by MOUD status. As shown in Table 2b, 22.7% of participants who reported currently taking methadone reported an overdose in the prior 6 months as compared to 40.8% who were on buprenorphine and 39.7% among those who were not currently taking methadone or buprenorphine. 75.0% of those taking buprenorphine reported daily or almost daily fentanyl or heroin use as compared to 80.3% among those taking methadone and 84.7% in participants not on MOUD. In the multivariable logistic regression model of overdose history (Table 3), methadone was associated with significantly reduced odds of a recent overdose (aOR = 0.49, 95% CI = 0.30–0.79) compared to participants who were not on MOUD. Daily or almost daily crack use was associated with greater odds of a recent overdose (aOR = 2.21, 95% CI = 1.44–3.39), and White, compared to Black participants, had significantly reduced odds of reporting a recent overdose (aOR = 0.52, 95% CI = 0.29–0.93) . For the proportion that had recently overdosed, the methadone group was significantly different from the no MOUD group (Chi Square = 11.2, *p* < 0.01). Frequency of heroin/fentanyl use, race, age, sex, and education level were not associated with a recent overdose.

## 4. Discussion

Among this sample of community recruited participants, those who reported current methadone use were much less likely to experience a recent overdose compared to those not on methadone or buprenorphine, which is consistent with prior research [30,31]. However, this was not the case with buprenorphine. There are several explanations for the finding that buprenorphine was not associated with overdose recency. It may be that compared with those who are not enrolled in MOUD, individuals prescribed buprenorphine have high levels of drug dependence and, hence, when they use illicit opioids, they ingest more drugs, which increases the chance of an overdose. We do not know if those who reported a recent overdose were on buprenorphine when the overdose occurred. It is possible that an overdose led to enrolling in a buprenorphine program. It is also likely that some individuals do not take buprenorphine as prescribed.

The causal pathway between buprenorphine use and overdose rates cannot be disentangled in this study. The lack of differences in overdose prevention behaviors between individuals on methadone and buprenorphine and those not taking methadone or buprenorphine may be due to several factors that cannot be determined in a cross-sectional study. However, these findings have clear implications regarding overdose and care prevention behaviors and recommendations for future research. Most individuals, regardless of MOUD status, did not report that they always had naloxone with them when they used opioids, and a large proportion did not go slow when using or first testing their drugs for potency. These findings highlight the urgent need for programs and spaces that facilitate overdose prevention behaviors.

Within this study sample of people who currently use opioids, approximately half of the participants reported current MOUD use. The rate of methadone use was approximately twice the rate of buprenorphine, which is a substantially higher ratio of burprenorphine use compared to earlier Maryland and US studies [32,33]. We found that a smaller proportion of Black compared to White participants were taking methadone. Future research should examine racial barriers to MOUD, which are likely due in part to social determinants of health. Notably, we did not recruit MOUD participants who were not using opioids and hence were unable to assess the frequency of use among all MOUD patients. However, these findings suggest that a substantial portion of MOUD patients continue to use opioids. Their continued contact with healthcare providers is an opportunity to promote training in behavioral overdose prevention and care skills. Some healthcare providers may view training in overdose prevention as militating against drug use cessation. However, there is no evidence to suggest that providing overdose prevention training increases drug use. Among opioid dependent individuals, illicit opioids may be used as a means to address withdrawal symptoms and cravings, which MOUD may not fully address. Future research should examine if those who are taking MOUD and overdose were taking MOUD at the time of their overdose. Also, research should examine how those on MOUD may be able to most effectively plan for opioid use, the skills needed for overdose prevention, and barriers to engaging in overdose prevention strategies.

These findings suggest the importance of diffusion of overdose prevention and care behaviors to those in MOUD and training people who use opioids to promote overdose prevention and care behaviors among their drug-using network members and others in the community. MOUD programs need to provide patients with the skills to train others in overdose prevention and care since naloxone is not self-administered, and others can also promote social norms of safer drug use behaviors. Moreover, as drug use is frequently a social behavior, there is a need to provide people who use opioids with the skillset necessary to develop harm reduction social norms and practices within their communities.

A focus on managing drug use and reducing the associated harms calls for an approach that acknowledges a wide range of trajectories of opioid and stimulant use and provides individuals with the training for risk reduction in a variety of situations. MOUD can be viewed as a medication to reduce the harms associated with opioid use as well as a medication to reduce harmful drug use. Some MOUD programs do provide naloxone [34,35,36]. However, MOUD programs should engage in more robust overdose prevention programs. Future research should develop programs that are tailored to both opioid treatment programs and healthcare providers who prescribe buprenorphine. Overdose prevention strategies address the amount and timing of drug ingested and the setting of use. Drug use settings can reduce fatal overdoses if naloxone is available and others at the setting are willing to administer it. The setting of use also may influence the amount and speed of use. In public or unsafe settings, there may be a rush to use, and hence, less attention is paid to safer use patterns [37,38]. Drug use settings may vary not only in terms of overdose but also for HIV/HCV risk due to sharing injection equipment or not adequately cleaning used equipment [37,39]. As the vast majority of individuals on MOUD will use illicit opioids while in treatment, in addition to providing naloxone, healthcare providers should help patients develop strategies to minimize HIV/HCV infection risks. Acknowledging that illicit opioid use is not a rare event for people on MOUD and promoting overdose prevention strategies within MOUD may help people who use opioids reduce their risk behaviors.

There are several study limitations that should be acknowledged. This was not a random sample, and the self-reports may have been influenced by social desirability bias. The study was also cross-sectional, which limits causal inferences. Moreover, as we sampled people who currently use opioids, we do not have information on those who are not currently using. Moreover, those who are attempting to cease drug use may be avoiding people who use drugs and places where drugs are frequently used; hence, they may have been less likely to be recruited due to avoiding areas of Baltimore where the recruiters were active. There are hopeful findings from this study. A large proportion of respondents had naloxone available when using drugs, and many people tested their drugs by ingesting them slowly.

## 5. Conclusions

These findings suggest that many people who use opioids in Baltimore are engaging in harm reduction strategies. However, there were no significant associations between methadone or buprenorphine status and overdose prevention and care behaviors. Unfortunately, even when harm reduction behaviors are common, there can be an exceedingly high rate of fatal overdoses within the community. Overdose risk could be reduced in many drug use episodes that involve opioids [40]. MOUD providers and public health entities need to urgently address the need to ensure that there are skills, settings, and resources for safer drug use and promote community social norms of safer drug use.

## Figures and Tables

**Table 1:** Demographic characteristics of study participants, Baltimore, Maryland, n = 647.

Question	Response	Frequency	Percent
Sex assigned at birth	Male	381	58.9
	Female	266	41.1
Self-reported race	Black	470	72.6
	White	142	21.9
	Other	35	5.4
Highest level of education	Grade 11 or less	188	29.1
	Grade 12 or GED	294	45.4
	Some college, Associates, Technical Degree	146	22.6
	Bachelor’s Degree or Greater	19	2.9
Employment status	Employed, full-time	21	3.2
	Employed, part-time	41	6.3
	Unemployed	273	42.2
	Retired	30	4.6
	Student	4	0.6
	Disabled/Unable to Work	278	43.0
In the past 6 months in prison or jail	Yes	17	2.6
Mental health condition such as depression, bipolar, or schizophrenia	Yes	451	69.7
Homeless in the past 6 months	Yes	280	43.3

**Table 2:** (**a**) The association between overdose prevention behaviors and MOUD status among study participants in Baltimore, Maryland (n = 647). (**b**) The association between overdose recency frequency of drug use and MOUD status among study participants in Baltimore, Maryland, (n = 647).

(**a**)						
**Overdose Prevention Behaviors**	**Drug Treatment Group**		**Always/Often** **n (%)**	**Sometimes** **n (%)**	**Rarely/Never** **n (%)**	**Chi-Square Value, *p*-Value**
Use a small dose first to test drug potency	No methadone or Buprenorphine		216 (64.7)	48 (14.4)	70 (21.0)	1.40, *p* = 0.85
	Methadone		134 (62.9)	36 (16.9)	43 (20.2)	
	Buprenorphine		66 (66.0)	17 (17.0)	17 (17.0)	
Use fentanyl test strips	No methadone or Buprenorphine		31 (9.3)	44 (13.2)	259 (77.5)	3.55, *p* = 0.47
	Methadone		30 (14.1)	31 (14.6)	152 (71.4)	
	Buprenorphine		11 (11.0)	14 (14.0)	75 (75.0)	
Go slow to check drug’s strength	No methadone or Buprenorphine		184 (55.1)	72 (21.6)	78 (23.4)	7.06, *p* = 0.13
	Methadone		115 (54.0)	36 (16.9)	62 (29.1)	
	Buprenorphine		58 (58.0)	25 (25.0)	17 (17.0)	
Use drugs alone	No MOUD		162 (48.5)	107 (32.0)	65 (19.5)	5.63, *p* = 0.23
	Methadone		97 (45.5)	58 (27.2)	58 (27.2)	
	Buprenorphine		46 (46.0)	27 (27.0)	27 (27.0)	
When using drugs, how often have Narcan			**Always** **Frequency (%)**	**Often/Sometimes** **Frequency (%)**	**Rarely/Never** **Frequency (%)**	8.94, *p* < 0.10
	No methadone or Buprenorphine		140 (41.9)	109 (32.6)	85 (25.4)	
	Methadone		102 (47.9)	70 (32.9)	41 (19.2)	
	Buprenorphine		36 (36.0)	45 (45.0)	19 (19.0)	
(**b**)						
**Drug Treatment Group**	**Last Time Overdose** **n (%)**		**Chi-Square** **Value, *p*-Value**	**Daily Use of Heroin/Fentanyl** **n (%)**		**Chi-Square Value, *p*-Value**
	More than 6 months ago	Last six months	Last six months	No	Yes	5.35, *p* < 0.10
Not currently taking methadone or buprenorphine	129 (60.3)	85 (39.7)	13.92, *p* < 0.001	51 (15.3)	283 (84.7)	
Methadone	126 (77.3)	37 (22.7)		42 (19.7)	171 (80.3)	
Buprenorphine	42 (59.2)	29 (40.8)		25 (25.0)	75 (75.0)	

**Table 3:** Correlates of reporting a drug overdose in the prior six months compared to participants who overdosed more than six months ago in Baltimore, Maryland (n = 448).

Overdose in the Prior Six Months		
Variables	OR (95% CI)	aOR (95% CI)
Currently taking methadone or buprenorphine (ref: no)		
Methadone	**0.45** **(0.28–0.70)**	**0.49** **(0.30–0.79)**
Buprenorphine	1.05 (0.61–1.81)	1.01 (0.57–1.80)
Daily or almost daily use of heroin or fentanyl	0.78 (0.47–1.29)	0.66 (1.18–0.38)
Daily or almost daily use of crack	**2.09** **(1.14–3.11)**	**2.21** **(1.44–3.39)**
Homeless in prior 6 months	1.47 (0.99–2.18)	1.55 (0.99–2.44)
Sex at birth	0.97 (0.65–1.45)	0.97 (0.63–1.49)
Age (continuous)	1.00 (0.98–1.01)	1.00 (0.98–1.02)
Race (ref: Black)		
White	0.66 (0.41–1.06)	**0.52** **(0.29–0.93)**
Other race	0.58 (0.24–1.40)	0.45 (0.18–1.15)
Level of education (continuous)	0.90 (0.71–1.15)	0.91 (0.71–1.18)

Bold = *p* < 0.05, OR = Odds Ratio, aOR = Adjusted Odds Ratio, CI = Confidence Interval.

## Data Availability

The data presented in this study are available on request from the corresponding author. Due to reports of illegal behaviors, the data will be unidentified.
